# Structural Electronic Skin for Conformal Tactile Sensing

**DOI:** 10.1002/advs.202304106

**Published:** 2023-09-22

**Authors:** Sen Li, Jiantao Huang, Meilan Wang, Ka Deng, Chenhui Guo, Bin Li, Yu Cheng, Hongyan Sun, Hong Ye, Tingrui Pan, Yu Chang

**Affiliations:** ^1^ School of Biomedical Engineering University of Science and Technology of China Hefei 230026 China; ^2^ Center for Intelligent Medical Equipment and Devices Suzhou Institute for Advanced Research University of Science and Technology of China Suzhou 215123 China; ^3^ Bionic Sensing and Intelligence Center (BSIC) Institute of Biomedical and Health Engineering Shenzhen Institute of Advanced Technology Chinese Academy of Sciences Shenzhen Guangdong 518055 China; ^4^ School of Engineering Hangzhou Normal University Hangzhou Zhejiang 311121 China; ^5^ TacSense Technology (Shenzhen) Co., Ltd Shenzhen Guangdong 518000 China; ^6^ Department of Precision Machinery and Precision Instrumentation University of Science and Technology of China Hefei 230026 China

**Keywords:** 3D printing, conformal tactile sensing, electronic skin, iontronic

## Abstract

The conformal integration of the electronic skin on the non‐developable surface is in great demand for the comprehensive tactile sensing of robotics and prosthetics. However, the current techniques still encounter obstacles in achieving conformal integration of film‐like electronic skin on non‐developable surfaces with substantial curvatures for contact pressure detection and tactile mapping. In this paper, by utilizing the 3D printing technology to prepare the 3D electrode array in the structural component following its surface curvature, and covering it with a molded functional shell to form the pressure sensitive iontronic interface, a device is proposed to achieve high‐sensitive pressure detection and high‐fidelity tactile mapping on a complicated non‐developable surface, called structural electronic skin (SES). The SES is prepared in a 3D printed fingertip with 46 tactile sensing units distributed on its curved surface, achieving the integration of both structural and tactile functions in a single component. By integrating the smart fingertip into a dexterous hand, a series of demonstrations are presented to show the dead‐zone free pressure detection and tactile mapping with high sensitivity, for instance, 2D pulse wave monitoring and robotic injection in a medical robot, object recognition and compliant control in a smart prosthesis.

## Graphical abstract

A structural electronic skin (SES) for conformal tactile sensing is proposed, which enables highly sensitive pressure detection and high‐fidelity tactile mapping on complex non‐developable surfaces. This structural electronic skin is successfully integrated into dexterous hands, enabling sensing units to completely cover non‐developable surfaces in a conformal manner, demonstrating its application in the fields of medical robotics and smart prosthetics.

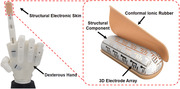

## 1 Introduction

Perception of tactile information can be of critical importance for humanoid robots and smart prosthetics, which has led to significant research interest in the essential component of tactile perception, namely electronic skin.^[^ ^1^ ^]^ Commonly, the electronic skin is considered as a membranous device combining the tactile perception function, such as contact pressure detection and tactile mapping, and the mechanical properties of flexibility.^[^ ^2^ ^]^ These smart films are mostly integrated with the robot via directly adhering to the surface.^[^ ^3^ ^]^ This assembly solution works well on developable surfaces, such as cylindrical surfaces, conical surfaces, etc., but faces problems on non‐developable surfaces.^[^ ^4^ ^]^ A non‐developable surface is one type of curved surface that cannot be developed into a plane, such as a spherical surface.^[^ ^5^ ^]^ Therefore, it is hard for the planar electronic skin film to fully cover the non‐developable surface in a conformal manner, leading to the loss of tactile function in some areas of the surface.^[^ ^6^ ^]^ However, humanoid robots or prosthetics may have abundant non‐developable surfaces that pose a challenge.^[^ ^7^ ^]^ For example, a fingertip can be considered as a non‐developable surface formed by joining a hemispherical surface with a cylindrical surface, which cannot be fully covered by adhering an electronic skin conformally.^[^ ^6^ ^]^ However, the tactile perception from the whole surface of the fingertip is of critical importance for daily interactions. For instance, knocking, grasping, and clamping the objects require tactile perception at the top, front, and side faces of the fingertip, respectively.^[^ ^8^ ^]^ In summary, the conformal integration of the electronic skin on a non‐developable surface is in great demand for the comprehensive tactile sensing of robotics and prosthetics.^[^ ^9^ ^]^

As for the challenges mentioned above, researchers have made corresponding attempts to address the problem. Currently, there are mainly four different strategies to cover electronic skins on non‐developable surfaces conformally.^[^ ^1^, ^9^ ^]^ The first one is to utilize the stretchability of the electronic skin to deform its shape along the curvature of the non‐developable surface.^[^ ^5^ ^]^ For instance, Chen and coworkers fabricated an electronic skin with a kirigami structure that conformably fits over the radial artery region of the human hand through stretching.^[^ ^10^ ^]^ Wang and coworkers have proposed a large‐area, elastomeric electronic skin covering the robotic hand for multifunctional detection, with five multifunctional sensing units on the fingertips and 15 pressure sensing units distributed over the finger phalanxes and palm.^[^ ^11^ ^]^ However, limited by the stretchability of the electronic skin, it is difficult to conformally attach the electronic skin to a non‐developable surface with a large curvature, for instance, the whole spherical surface, using this approach currently.^[^ ^12^ ^]^ The second method uses traditional coating techniques, such as brush painting or spray coating, to generate electronic skin on the surface.^[^ ^13^ ^]^ For example, Park and coworkers fabricated an electronic skin on a robotic hand via spraying resistive coating to detect bending motion.^[^ ^14^ ^]^ However, this technique is challenging to realize the sensing arrays on the 3D surfaces because it does not involve any patterning process, limiting this method to achieve tactile mapping, which is very important for object recognition based on tactile perception.^[^ ^15^ ^]^ The third one uses the additive patterning process to generate electronic skin on 3D surfaces, such as direct printing.^[^ ^16^ ^]^ In this method, the height and angle of the printing nozzle are controlled to follow the curved surface while printing functional materials.^[^ ^17^ ^]^ Chung's group has printed self‐healing hydrogel to prepare an electronic skin conforming to the shape of the finger to achieve the full coverage of the target surface with this functional material.^[^ ^4c^ ^]^ McAlpine's group has developed an adaptive 3D printing technology to print functional materials directly onto moving freeform surfaces.^[^ ^18^ ^]^ However, the method to conformally print functional materials on a non‐developable surface requires a complicated mechanical system and algorithm to guarantee the appropriate distance and angle between the printing nozzle and the curved surface. Moreover, the printing of a multilayer circuit on a curved surface, of which extremely‐high precision mechanical control and multi‐materials printing are required, is still challenging currently, while a multilayer circuit is of importance for the construction of high‐resolution sensing array to achieve high‐fidelity tactile mapping.^[^ ^18^ ^]^ The final solution is to partition the non‐developable surface into smaller regions, each of which can be regarded as developable surfaces, thereby achieving an approximate development of the entire surface. This approach is exemplified by the classical method to develop a spherical surface into an array of fusiform surfaces.^[^ ^19^ ^]^ For instance, Lu and coworkers described a high‐density and hemispherically curved image sensor array based on the truncated icosahedron design (fullerene‐like structure) that conform well to an eyeball without any stretchable structures.^[^ ^20^ ^]^ Huang and coworkers proposed a kirigami assembly strategy for conformal electronics that involves cutting a 2D sheet into small, kirigami‐patterned pieces using a 2D‐to‐3D mapping algorithm and then reassembling them onto a prescribed 3D curved surface.^[^ ^21^ ^]^ However, it's worth noting that this conformal approximation method may result in some areas being left uncovered, particularly on complex non‐developable surfaces. Moreover, the irregular shape of the approximated developable surface significantly increases the complexity of circuit design. In conclusion, it is still challenging for current methods to realize the conform integration of the electronic skin on the non‐developable surface with a large curvature for force sensing and tactile mapping.

3D printing has garnered significant attention for its ability to efficiently and cost‐effectively produce intricate 3D components.^[^ ^22^ ^]^ Thus far, a wide range of 3D printing technologies have been employed in the manufacturing of various functional devices with complicated internal structures and complex geometries, including energy storage devices, mechanical sensors, biomedical implants, etc.^[^ ^23^ ^]^ Therefore, 3D printing has the potential to produce multi‐layer circuits that conformally adhere to or are embedded within printed components with non‐developable surfaces.

**Figure 1:**
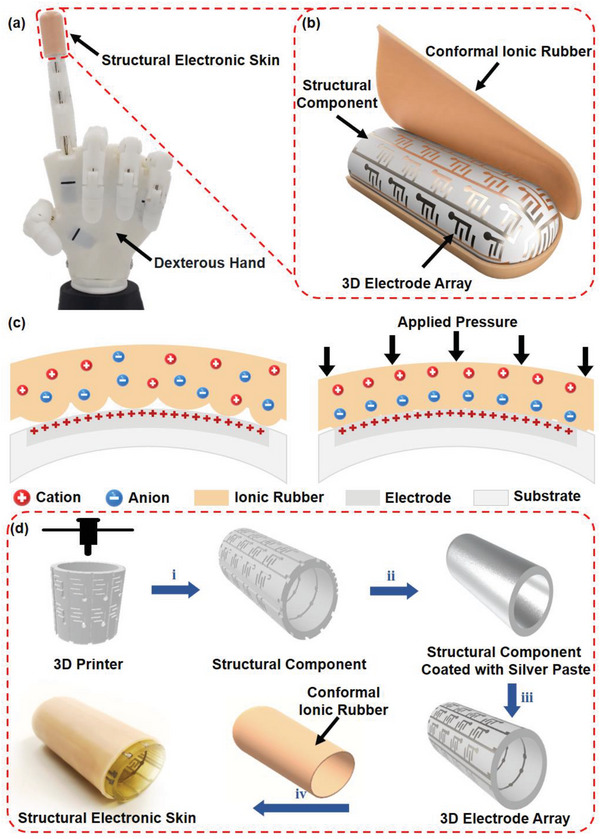
a) The photo of the structural electronic skin (SES) integrated with a dexterous hand; b) the structure of the SES; c) the pressure sensing mechanism of the SES; d) the flow chart of the preparation process of the SES, i, ii, iii, and iv refer to 3D printing, silver paste coating, remove of redundant silver paste and device assembly respectively.

By utilizing 3D printing technology to prepare a multi‐layered circuit embedded in the structural component and combining it with molded ionic silicon rubber to form a pressure‐sensitive iontronic interface, we have proposed a device called Structural Electronic Skin (SES) that can achieve high‐sensitivity force detection and high‐fidelity tactile mapping on complicated non‐developable surfaces, as shown in **Figure** **1**a,b. The sensing principle of SES is based on an iontronic mechanism, where an ionic elastomer conformally covers a 3D interdigital electrode array. Specifically, the 3D interdigital electrode array is prepared in a customized 3D printed structural component following its surface curvature. Notably, the electrode array is designed into a double‐layer circuit through metallized through holes, greatly decreasing the complexity of the circuit layout for tactile mapping. Furthermore, a moldable composite of PDMS with high ionic conductivity and elasticity is cast into a conformal shell that matches the shape of the 3D printed structural component, known as conformal ionic rubber, to provide complete coverage of the 3D interdigital electrode array. Significantly, the internal surface of the conformal ionic rubber exhibits a coarse texture that facilitates the development of a pressure‐sensitive interface. As a result, the SES devices have demonstrated a remarkable sensitivity of up to 3.59 nF/kPa/cm^2^, achieving a single‐Pascal pressure resolution of 8.6 Pa, a mechanical response of tens of milliseconds, and an adjustable measurement range of up to 500 kPa. Furthermore, the SES is prepared in a 3D printed fingertip with 46 tactile sensing units distributed on its curved surface. By integrating the smart fingertip into a dexterous hand, a series of demonstrations have been presented to show the dead‐zone free pressure detection and tactile mapping with high sensitivity, for instance, 2D pulse wave monitoring and robotic injection in a medical robot, object recognition and compliant control in a smart prosthesis.

## 2 Results and Discussion

### 2.1 Mechanical Response Principle of the SES

The mechanical response of the SES is derived from the pressure sensitive iontronic interface between the 3D interdigital electrode and the conformal ionic rubber. As shown in Figure 1c, the external pressure applied to the sensing unit leads to the mechanical deformation of the micro‐structure on the conformal ionic rubber, resulting in an increase in the contact area of the iontronic interface and the EDL capacitance of the sensing unit. Commonly, the mechanical response of the iontronic pressure sensor is highly related to the mechanical and electrical properties of the functional materials for a given sensing structure.^[^ ^24^ ^]^ Specifically, the capacitance‐pressure curve of the SES unit is a function of Young's modulus, unit area capacitance (UAC), and surface morphology of the conformal ionic rubber. According to the literature, for a given surface morphology, lower Young's modulus and higher UAC of functional materials result in larger capacitive output at a certain pressure, i.e., higher sensitivity of the device, while higher Young's modulus leads to wider linear response range, i.e., larger sensing range. Since the conformal ionic rubber is prepared using the same template, the performance control of the SES device mainly focuses on the adjustment of the ionic rubber.^[^ ^25^ ^]^ The SEM images of the ionic rubber surface and the mold surface are shown in Figure S1 (Supporting Information).

### 2.2 Preparation Process of the SES

For a better understanding of the architecture of the SES, a brief flow chart of the preparation process is shown in Figure 1d. First, an FDM 3D printer is used to construct a hollow structural component, leaving grooves and through holes on the surface for circuit building. Afterward, silver paste is coated on the structural component to fill all the grooves and through holes. After curing, redundant silver paste on the surface is removed by sanding, remaining conductive patterns in the grooves and through holes to form the 3D electrode array. Finally, the SES can be obtained by assembling the 3D electrode array and the conformal ionic rubber, which is cast into a shell in accordance with the shape of the 3D printed structural component. The detailed preparation process of SES is shown in Figure S2 (Supporting Information). Figure S3 (Supporting Information) demonstrates the elasticity of ionic rubber.

### 2.3 Material Properties of the Ionic Rubber

#### 2.3.1 Ion Conducting Model of Ionic Rubber

The preparation of the ionic rubber is the key of this work. In order to achieve a castable elastomer with high ionic conductivity, an ionic PDMS is developed. Compared with the most commonly used ionic elastomer, i.e., ionic gel, PDMS always possesses a higher recovery, stability, and heat resistance.^[^ ^26^ ^]^ As a thermosetting resin, PDMS can be cast into the mold at room temperature and cured rapidly with heat, while most gels cannot be cast for the evaporation of the solvent during curing or require complicated curing approaches, for instance, UV curing, repeated freezing, etc.^[^ ^27^ ^]^ Furthermore, the extremely low surface energy of silicone rubber makes it easy to be released from the mold.

**Figure 2:**
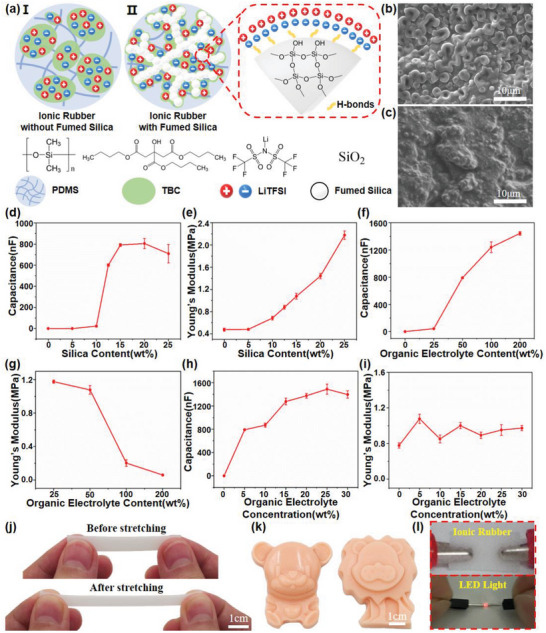
a) Schematic diagram of the internal structure of ionic rubber I without and II with fumed silica added, and the chemical composition of the ionic rubber; the SEM images of the cross section of the ionic rubber b) without and c) with fumed silica added; d) the UAC of the ionic rubber with different fumed silica contents; e) Young's modulus of the ionic rubber with different fumed silica contents; f) the UAC of the ionic rubber with different organic electrolyte contents; g) Young's modulus of the ionic rubber with different organic electrolyte contents; h) the UAC of the ionic rubber with different organic electrolyte concentrations; i) Young's modulus of the ionic rubber with different organic electrolyte concentrations; j) the photos of ionic rubber before and after stretching; k) photos of ionic rubber cast into various 3D shapes; l) the photo to demonstrate the conductivity of the ionic rubber.

The ionic modification of PDMS requires the addition of ion conducting materials, especially liquid ionics for their high ionic conductivity, into the PDMS matrix. However, liquid ionics always show poor compatibility with PDMS for their big difference in polarity.^[^ ^28^ ^]^ The liquid ionics tend to disperse in the PDMS in the form of micro‐droplets, which fails to generate a complete network for ion migration, as shown in image I in **Figure** **2**a. The SEM image of the liquid ionics mixed PDMS in Figure 2b also represents the morphology of the surface with abundant independent cavities with a diameter of ≈1 µm. As a result, the liquid ionics mixed PDMS showed an extremely low UAC of dozens of pF cm^−2^, which is about the same with pure PDMS, and no matter how much liquid ionics are added, the ions cannot migrate freely in the material, limiting the sensitivity of SES. In order to solve this problem, fumed silica, one kind of silica particle about several nanometer in diameter, is added into the system to improve the dispersion of liquid ionics in PDMS. The fumed silica contains abundant polar groups, such as hydroxy groups, on the surface, which can adsorb liquid ions with similar polarity via opposite charges and hydrogen bonds.^[^ ^29^ ^]^ Fumed silica can be easily dispersed in PDMS through stirring and can form an interconnected network when its concentration reaches the percolation threshold.^[^ ^30^ ^]^ Therefore, the adsorbed liquid ionics on the surface can also form a complete network for ion migration, as shown in image II in Figure 2a. The SEM image of the ionic PDMS with fumed silica in Figure 2c demonstrates a homogeneous morphology without any visible cavities, indicating the uniform dispersion of the liquid ionics in the PDMS. Meanwhile, the measured UAC exhibits an increase of several orders of magnitude to hundreds of nF cm^−2^, significantly improving the sensitivity of the SES for slight pressure detection.

#### 2.3.2 Mechanical and Electrical Properties of the Ionic Rubber

The ionic PDMS rubber is mainly composed of three components, which are PDMS polymer, liquid ionics, and fumed silica. Here, an organic electrolyte prepared by dissolving bistrifluoromethanesulfonimide lithium salt (LiTFSI) into tributyl citrate (TBC), a hydrophobic solvent with a high boiling point of 225 °C, is chosen as the liquid ionics for its low cost compared with ionic liquid and high stability in the atmosphere compared with water. This section focuses on the researches between the ratio of the components and the properties of the ionic PDMS rubber for performance optimization. The first component to be studied is fumed silica. As mentioned above, the amount of fumed silica in the system will influence the UAC of the ionic rubber dramatically. Figure 2d has summarized the UAC of the ionic rubber with different fumed silica content. Ionic rubbers with low fumed silica content of 0% and 5% (compared with the weight of PDMS polymer) demonstrate extremely low UACs of 10.2 pF cm^−2^ and 15.4 pF cm^−2^. When the proportion of fumed silica increases to 10%, the UAC rises to 22.8 nF cm^−2^, which is more than 1000 times higher than that of 5% before. When the proportion of fumed silica in ionic rubber reaches the “percolation threshold”, the fumed silica particles with organic electrolytes adsorbed on the surface are interconnected to form a complete ionic conducting network, significantly increasing the UAC of the ionic rubber. Continue to increase the proportion of fumed silica to 12.5%, the UAC increases sharply to 601.2 nF cm^−2^, revealing that the “percolation threshold” content of fumed silica in ionic rubber is ≈10% to 12.5%. When the proportions of fumed silica increase to 15%, 20%, and 25%, the UACs can reach 791.3, 805, and 709.4 nF cm^−2^, respectively. It is evident that the UAC of the ionic rubber tends to be stable when the fumed silica content exceeds 15%. Therefore, the optimized fumed silica content in the ionic rubber is decided to be 15% to achieve the highest UAC. Another noteworthy property is Young's modulus of the ionic rubber according to the theoretical analysis of the mechanical response principle of the SES. The influence of fumed silica content on Young's modulus of ionic rubber is shown in Figure 2e. As can be seen, with the continuous increase of fumed silica content from 0% to 25%, the Young's modulus of the ionic rubbers increases from 0.48 to 2.18 MPa accordingly, because the hard solid pigment will limit the movement of polymer segments, leading to the advance of the Young's modulus of the material. Figure 2f,g summarizes the influences of liquid ionics content on the properties of the ionic rubber. It is shown that the liquid ionic content demonstrates a positive influence on the UAC of the ionic rubber, while the Young's modulus decreases as the liquid ionic content rises for the liquid in the polymer will promote the segment movement.^[^ ^31^ ^]^ An optimized liquid ionics content is decided to be 50% (compared with the weight of PDMS polymer), for a higher content will cause the exudation of the liquid ionics under pressure. The concentration of LiTFSI in the liquid ionics will also affect the UAC of the ionic rubber, as shown in Figure 2h, the UAC of the ionic rubber increases with the LiTFSI concentration. However, the optimized concentration of LiTFSI in the liquid ionics has been determined to be 10% (compared with the weight of liquid ionics), considering the high cost of LiTFSI and the long‐time dissolving process. Finally, Figure 2i demonstrates that the Young's modulus of the ionic rubber is minimally affected by variations in LiTFSI concentration. As a result, the optimized formula for the ionic rubber has been determined to consist of 15% fumed silica and 50% liquid ionic with a LiTFSI concentration of 10% in TBC, taking into account the UAC, stability, and cost of the ionic rubber. In the aforementioned experiment, each parameter was subjected to five tests to obtain the average value, and the corresponding standard deviation was calculated accordingly.

#### 2.3.3 Stretchability, Castability and Ionic Conductivity of Ionic Rubber

To visually demonstrate the mechanical and electrical properties of the ionic rubber, a series of tests have been conducted. The stretchability of the ionic rubber with an optimized formula is demonstrated in Figure 2j, where it remains intact even after being stretched by 50% and can be restored to its original state upon unloading the tensile force. Furthermore, the ionic rubber can be cast into various 3D shapes, similar to pure PDMS, as illustrated in Figure 2k. To test the conductivity of the ionic rubber, connect it in series with an LED light and apply an AC signal with a frequency of 1 kHz and a voltage of 30 V. The circuit diagram is shown in Figure S4a (Supporting Information), and the experimental setup is shown in Figure S4b (Supporting Information). The result shows that the LED light can be illuminated, which indicates that the high ionic conductivity of the ionic rubber, as shown in Figure 2l.

### 2.4 Properties of the 3D Electrode Array

#### 2.4.1 Preparation of the Multi‐Layer Conductive Patterns in a 3D Printed Component

**Figure 3:**
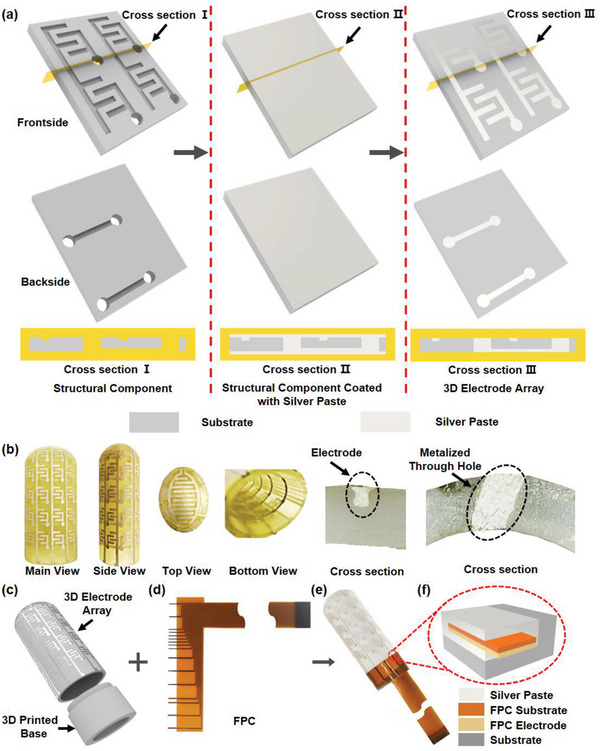
a) Flow chart of the 2 × 2 3D electrode array preparation process; b) photos of the 3D electrode array in different views and the microscopic images of cross section of electrode and metalized through hole; c) package exploded view of the 3D electrode array; d) photo of flexible printed circuit (FPC) for bonding the 3D electrode array with the signal acquisition circuit; e) photo of 3D electrode array bonded with FPC; f) the schematic diagram of the bonding areas.

The preparation of multi‐layer conductive pattern on a 3D printed component is the key to realize conformal sensing of SES. In order to express the preparation process of the multi‐layer conductive pattern more clearly, the frontside view, backside view, and cross section view of a 2 × 2 3D electrode array are demonstrated in **Figure** **3**a. First, the 3D structural component with patterned grooves and through holes was fabricated by an FDM 3D printing process, where the grooves and through holes are used for building the multi‐layer conductive patterns as the sensing electrode. Here, the sensing electrode is designed into an interdigital electrode array, where the electrodes of each column are wired on the outside surface of the 3D printed component, while the electrodes of each row are connected to the circuit at the backside via the through holes. Afterward, conductive silver paste is coated on the structural component to fill all the grooves and through holes, followed by heat treatment to cure the silver paste, as shown in cross section II in Figure 3a. Finally, redundant silver paste on the surface is removed by sanding, remaining conductive patterns in the grooves and through holes to form the 3D electrode array. The photos in Figure 3b illustrate the high quality conformal conductive patterns in the 3D printed component, of which the cross section view of the 3D electrode array shows a complete metalized through holes for the electrical connection of multi‐layer circuit. According to the preparation process discussed above, the 3D electrode array of the SES can be prepared in the 3D printed components with various shapes conformally, such as a fingertip with abundant non‐developable curves, as shown in Figure 3c.

#### 2.4.2 Flexible‐Rigid Bonding of the 3D Electrode Array

In order to connect the 3D electrode array with the signal acquisition circuit, a flexible printed circuit (FPC) is used as the interconnector, as shown in Figure 3d. The schematic diagram of flexible printed circuit design is shown in Figure S5 (Supporting Information). Here, all the column and row circuits of the 3D electrode array gather to the bonding pads at the bottom edge of the 3D printed component. Afterward, the FPC interconnector is cut by laser to protrude the bonding areas, as shown in Figure 3c. After being wrapped on a 3D printed base which can be inserted into the hollow 3D printed component for fixation, the protruded bonding areas of the FPC are placed on the bonding pads to achieve the electrical connection using conductive silver paste, as shown in the photos in Figure 3e and the schematic diagram in Figure 3f.

### 2.5 The Sensing Performances of the SES

#### 2.5.1 The Capacitance‐To‐Pressure Properties of the SES

**Figure 4:**
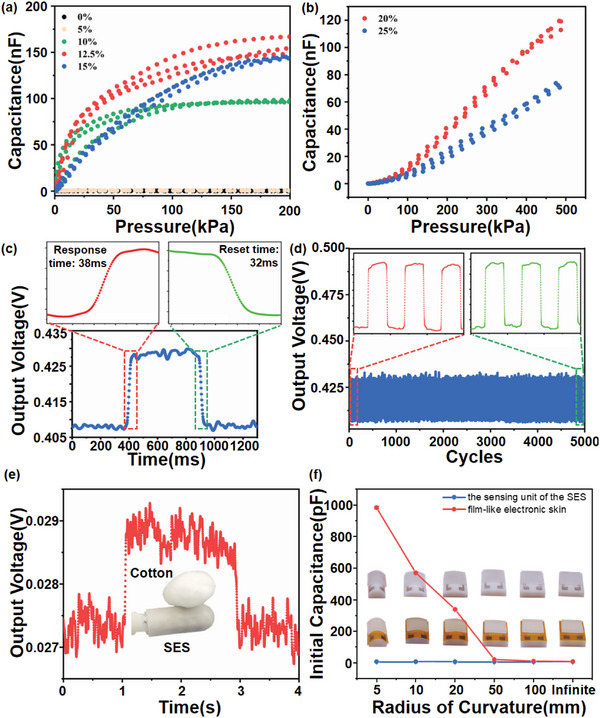
a) The capacitance‐to‐pressure (C–P) curves of sensing units of the structural electronic skins using the ionic rubbers with different fumed silica contents (from 0% to 15%); b) C–P curves of sensing units of the structural electronic skins using the ionic rubbers with different fumed silica contents (20% and 25%) under 500 kPa; c) response time and reset time of the sensing unit of the structural electronic skin; d) the output stability of the sensing unit of the structural electronic skin under cyclic mechanical loads of 10 Hz for 5000 cycles; e) the measured output voltage curve when loading and unloading ultralow pressure applied by a cotton; f) the initial capacitance of the sensing unit of the structural electronic skin and film‐like electronic skin under different radius of curvature.

In order to further evaluate and optimize the sensing performance of the SES, we configured the sensing unit of the SES with a sensing area of 1 cm^2^ for testing. The capacitance‐pressure curve is one of the most critical performances of the SES, from which key device parameters such as sensitivity and sensing range can be deduced. According to the theoretical analysis, the sensitivity and the sensing range of the SES are related to the Young's modulus and UAC of the ionic rubber, both of which can be adjusted by the fumed silica contents in the ionic rubber. Theoretically, the sensitivity of SES devices is positively correlated with UAC but negatively correlated with Young's modulus of functional materials, so the relationship between the content of fumed silica in ionic rubber and SES sensitivity is still uncertain. However, the sensing range is positively correlated with the Young's modulus of the ionic rubber, so increasing the fumed silica contents leads to a larger sensing range theoretically. The experimental results of the capacitive‐pressure response curves of the sensing units using the ionic rubber with different fumed silica contents are illustrated in **Figure** **4**a (fumed silica contents from 0% to 15%) and Figure 4b (fumed silica contents from 20% to 25%). Specifically, sensing units using ionic rubber with fumed silica content of 0% and 5% exhibited extremely low capacitance increase under external pressure loading, with average pressure sensitivities below 200 kPa of only 0.071 pF/kPa/cm^2^ and 0.23 pF/kPa/cm^2^ respectively, because the inadequate fumed silica in the ionic rubber cannot form the conductive networks. When the proportion of fumed silica in the ionic rubber increases to 10%, 12.5%, and 15%, the corresponding sensing units exhibit significantly increased sensitivity of 1.79, 3.59, and 1.52 nF/kPa/cm^2^ respectively below 25 kPa. Although the sensing unit using the ionic rubber with 12.5% fumed silica content represents the highest sensitivity, the one using the ionic rubber with 15% fumed silica content shows a larger sensing range of 200 kPa. Further increasing the fumed silica content to 20% and 25% will advance the linear sensing ranges to more than 500 kPa, as shown in Figure 4b, but decreases the sensitivities to 0.24 and 0.15 nF/kPa/cm^2^ within the linear sensing range of 500 kPa. In summary, the sensitivity and sensing range of the SES can be simply controlled by adjusting the sensing material to meet specific demands. Considering the comprehensive performance of the SES with high sensitivity and sensing range, the fumed silica content in the ionic rubber is optimized to be 15%.

#### 2.5.2 The Response Time, Repeatability, and Minimal Pressure Resolution of the SES

Rapid mechanical response is critical for electronic skin to process external mechanical stimuli quickly and effectively. Here, a piezoelectric actuator driven by a square wave signal is used to apply a periodic mechanical load to the sensing unit of the SES. The output signal from the structural electronic skin is processed by the amplifier circuit into a voltage signal and recorded by the data acquisition card at a sampling rate of 1 kHz, as seen in Figure S6 (Supporting Information) for the experimental setup. The output voltage signal of the amplifier circuit and the capacitance of the sensor exhibits a positive correlation, as depicted in Figure S7 (Supporting Information). Periodic loads were applied to the sensing unit at a frequency of 1 Hz to test the response time and reset time. As illustrated in Figure 4c, the response time of 38 ms and reset time of 40 ms can be extracted from a single pressure cycle. The fast response time and reset time ensure accurate sensing of the structural electronic skin under rapidly varied pressure, demonstrating the device's potentially superior performance in monitoring high‐frequency mechanical signals. The repeatability and stability of the structural electronic skin were tested using periodic loads at a frequency of 10 Hz. As shown in Figure 4d, the initial output voltage without pressure load and the peak output voltage under pressure load remained almost unchanged over 5000 pressure cycles, indicating the high stability and repeatability of the structural electronic skin. Taking advantage of the high noise immunity of the iontronic sensing mechanism, the sensing unit of the SES exhibited ultralow detection limitation, as shown in Figure 4e. Experimental setup can be found in Figure S8 (Supporting Information). Single pascal (≈8.6 Pa) level detection of ultralight objects has been successfully demonstrated (e.g., cotton of 0.086 g placed on a structural electronic skin sensing unit with 1.0 × 1.0 cm^2^ sensing area).

#### 2.5.3 Bending Insensitivity of the SES

Bending always cause stress between the layers of flexible pressure sensors, so bending insensitivity is of essence to achieve high precision pressure sensing. Current film‐like electronic skins will inevitably bend when they are adhered to a curved surface, introducing bending‐induced stress to the device. As shown in Figure 4f, the film‐like electrode skin prepared by assembling ionic rubber with FPC electrode has been bent over the surfaces with different radii of curvature. However, as the radii of curvature decrease below 20 mm, the capacitive outputs of the sensor increase rapidly, which has influenced the accuracy of pressure measurement. As a comparison, the sensing units of the SES with different radii of curvature all show zero output because no bending is needed when they are conformally prepared on the curved surfaces.

### 2.6 The Comparison with Current Literatures about Conformal Sensing

In order to illustrate the advantage of SES, a comprehensive comparison of the literatures about conformal sensing in terms of the number of sensing units, shape of the coverd surface, proccssing technology, sensing sensitivity, applications, conformal methods, etc. have been presented in **Table** **1**.

**Table 1:** Comparison of the literatures about conformal sensing.

	Number of sensing units	Shape of the covered surface	Processing technology	Full coverage or not	Sensing Sensitivity	Sensing mechanism	Applications	Conformal methods
ref. [1e]	16	Dorsal surface of finger	Laser processing and transfer printing	No	8053.1 kPa^−1^ (<1 kPa) 3103.5 kPa^−1^ (1–34 kPa)	Iontronic and triboelectric	Sign language cognition and robotic interaction	Stretching deformation
ref. [10]	1	Body artery sites	Laser cutting	No	35.2 mV Pa^−1^	Triboelectric	Wireless cardiovascular monitoring system and wearable electronics	Stretching deformation
ref. [11]	15	Human palm	Layer‐by‐layer Screen printing	No	0.025 V kPa^−1^	Piezoresistive	Wearable electronics	Stretching deformation
ref. [14]	1	Human joints	Spray coating and solution‐based process	No	Gauge factor of ≈56 at 70% strain	Piezoresistive	Robotic electronic skin or wearable electronic	Surface coating
ref. [4c]	1	Ring‐shaped and fingertip‐shaped	3D Printing	Yes	‐	Surface capacitive touch	Robotic electronic skin	Additive patterning process
ref. [18]	1	Back of hand	3D Printing	No	‐	‐	wearable devices on the body and for advanced medical treatments	Additive patterning process
ref. [20]	‐	Hemispherical surface	Chemical vapor deposition (CVD), photolithography, dry etching and lift‐off process	Yes	‐	Photovoltaic effect	Soft implantable optoelectronic device	Approximate development
ref. [21]	24	Hemispherical surface	Mechanical cutting and evaporation	Yes	‐	Capacitive	Conformal heater and a conformal wind sensing system	Approximate development
This Work	46	Fingertip	3D Printing, pouring and molding	Yes	3 nF/kPa/cm^2^	Iontronic	Medicine robot and smart prosthetics	Additive patterning process

## 3 Applications

Benefiting from the superior performances of the SES, such as high mechanical sensitivity, adjustable dynamic sensing range, on‐demand 3D fabrication, and excellent conformability, the sensor has been fabricated into a 3D printed fingertip with 46 tactile sensing units distributed on its curved surface. The electrode unit size of the 3D electrode array is 4 × 4 mm^2^, and the line width of the interdigitated electrodes is 0.5 mm. The capacitance‐to‐pressure response of the sensing unit based on the designed electrode size is presented in Figure S9 (Supporting Information). The smart fingertip can be integrated into a dexterous hand to deal with multiple application scenarios, such as 2D pulse wave monitoring and robotic injection as a medical robot, object recognition and compliant control as a smart prosthesis.

### 3.1 SES Embedded Medicine Robot for 2D Pulse Wave Monitoring and Robotic Injection

**Figure 5:**
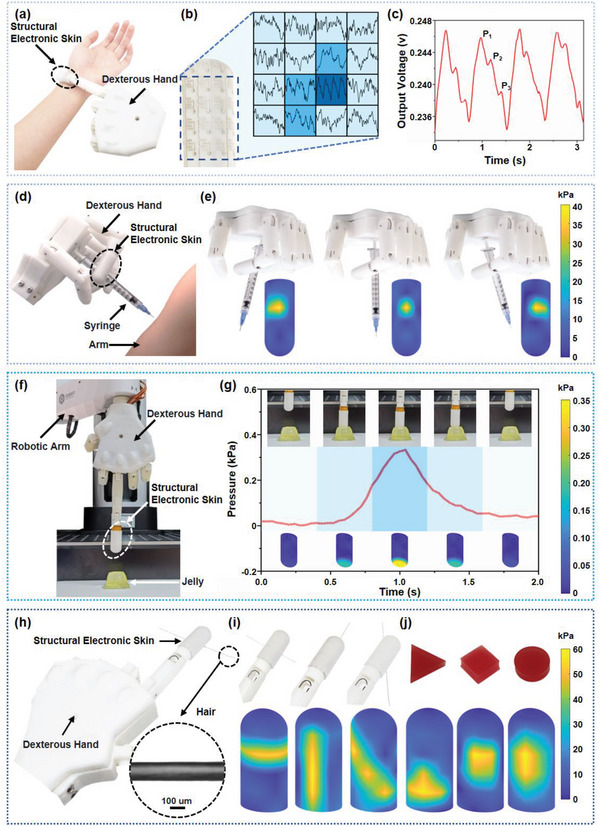
a) Photograph of structural electronic skin integrated on a dexterous hand to measure radial artery pulse; b) tactile measurement results of the radial artery pulse waveforms from a 4 × 4 sensing array on the front of the structural electronic skin; c) radial artery pulse waveform corresponding to the dark blue background extracted; d) photograph of robotic gripping of a syringe to simulate intramuscular injection; e) experimental results of structural electronic skin detection of the syringe grip state; f) the experimental setup for the compliant control experiment; g) experimental data and process of the compliant control experiment; h) photograph of structural electronic skin integrated on a dexterous hand to identify human hair; i) tactile mapping of structural electronic skin to recognize hair strands placed in different orientations; j) the tactile mapping of structural electronic skin to recognize objects of different shapes.

Medical robots have been widely used in the diagnosis and treatment of diseases due to their high efficiency, safety and precision.^[^ ^32^ ^]^ The future medical robot could achieve more complicated operations with the assistance of dexterous hands and robotic tactile in a humanoid mean. For instance, pulse wave detection is of great significance for cardiovascular disease diagnosis and monitoring because it can reflect multiple related physiological indices, such as heart rate, heart rate variability, blood pressure, pulse wave velocity, etc. The accuracy detection of the pulse wave requires the sensor to be placed on the superficial artery, while single point pressure sensor always need multiple attempts to find the appropriate location. However, the 2D pulse wave can solve the problem by recording the pulse waveforms covering a certain area around the superficial artery in an array form simultaneously, because at least one unit of the array can obtain the accurate pulse wave. To demonstrate the clinical application of the SES in robotic medicine, the SES embedded fingertip is placed on the radial artery of a healthy volunteer hand to monitor 2D pulse waves, as illustrated in **Figure** **5**a. Figure 5b summarizes the detected 2D pulse waveforms array from the 4 × 4 sensing units in the front surface of the smart fingertip, from which the dark blue unit represents the pulse wave with the highest accuracy and precision. By amplifying the waveform of the dark blue unit, three distinguishable characteristic peaks, namely the systolic peak (P1), the reflected systolic peak (P2), and dicrotic peak (P3), can be clearly distinguished, which can be further used for real‐time quantitative assessment of relevant hemodynamic parameters such as arterial stiffness and blood pressure, as shown in Figure 5c.

Non‐contact operations are essential to prevent disease transmission between doctors and patients. With the development of robotic technology, medicine robots are expected to replace humans in more medical operations with the risk of infection. Taking injection as an example, robotic injection can avoid direct contact between doctors and patients and improve injection efficiency. However, robotic injection requires sensors to detect the angle of the syringe to be injected into the skin for damage prevention. Here, the SES embedded smart fingertip can detect the angle of the syringe held between the index and middle fingers using the sensing units distributed on the side of the fingertip, as shown in Figure 5d. Specifically, the pressure heat map of the smart fingertip can reflect the contact angle between the fingertip and the syringe. As shown in Figure 5e, when the syringe is in a vertical position, the contact region between the syringe and the smart fingertip is mainly located in the center of the side of the fingertip, while the pressure heat map illustrates a distinct dot in the center unit. As the syringe tilts left or right, the contact region between the fingertip and the syringe varies, and the adjacent units near the center can also be influenced, with the pressure heat map illustrating obvious changes correspondingly.

### 3.2 SES Embedded Smart Prosthesis for Compliant Control and Object Recognition

Compliant control and object recognition are essential functions for smart prosthesis. The SES embedded dexterous hand could provide the capacities of contact pressure detection for compliant control and tactile mapping for object recognition, while maintaining a realistic appearance. Specifically, Figure 5f shows the experimental setup for the compliant control experiment. The SES embedded dexterous hand is mounted on a robotic arm (DOBOT M1) as an end effecter. The robotic arm was preprogrammed to go downward at a speed of 10 mm ^−1^s to bring the fingertip of the dexterous hand into contact with the extremely brittle jelly, while the contact pressure was recorded by the top sensing unit of the SES. Figure 5g demonstrates the photos, contact pressure curve, and pressure heat maps of the entire process. As can be seen, when the fingertip touches the jelly, the contact pressure increases correspondingly until reaching a predetermined threshold of 0.35 kPa, which is about the same pressure as placing a Ping‐Pong ball, and the robotic arm stops immediately and moves upward to its original position, avoiding the damage to the brittle jelly. A detailed video of the experiment can be found in the supplemental material. To demonstrate the ability of the SES on object recognition, the SES embedded dexterous hand is used to obtain the pressure heat map by pressing the objects with different shapes. For instance, benefiting from the high sensitivity, the SES can identify a human hair with a diameter of 100 µm from the pressure heat map, as shown in Figure 5h. The results of the acquired morphological information are summarized in Figure 5i, where the basic configuration and the orientation of the hair can be recognized in the pressure heat maps. Notably, the fingertip rotates from side to side when pressing to obtain an intact pressure heat map of the hair. In addition, the SES can also recognize several objects with different shapes. As shown in Figure 5j, when pressing the rubber blocks with triangular, square, and round shapes, the pressure heat maps demonstrate the corresponding shape, respectively.

Benefiting from the high mechanical sensitivity and conformal distribution of tactile sensing units across its entire surface, the smart fingertip has potential applications in monitoring 2D pulse waves and detecting syringe angles for disease diagnosis and treatment in medical robots, as well as achieving compliant control and object recognition in smart prosthetics.

## 4 Conclusion

In this paper, by utilizing the 3D printing technology to prepare the 3D electrode array in the structural component following its surface curvature, and covering it with a molded functional shell to form the pressure sensitive iontronic interface, we have proposed a device to achieve high‐sensitive pressure detection and high‐fidelity tactile mapping on a complicated non‐developable surface, called structural electronic skin (SES). Notably, the 3D electrode array is designed into a double‐layer circuit connected through metallized through holes, resulting in a significant reduction of complexity in the tactile mapping circuit layout. Moreover, a moldable PDMS composite with high ionic conductivity and elasticity is prepared for the first time and cast into the functional shell in accordance with the shape of the structural component to cover the 3D electrode array conformally. By adjusting the mechanical and electrical properties of ionic rubber, the performance of SES can be tailored to meet specific requirements in terms of sensitivity and sensing range. Consequently, the structural electronic skin devices have demonstrated a sensitivity of up to 3.59 nF/kPa/cm^2^, a single‐Pascal pressure resolution of 8.6 Pa, a mechanical response of tens of milliseconds, and an adjustable measurement range of up to 500 kPa. The SES is prepared in a 3D printed fingertip with 46 tactile sensing units distributed on its curved surface. By integrating the smart fingertip into a dexterous hand, a series of demonstrations have been presented to show the dead‐zone free pressure detection and tactile mapping with high sensitivity, for instance, 2D pulse wave monitoring and robotic injection in a medical robot, object recognition and compliant control in a smart prosthesis.

## 5 Experimental Section

### Preparation of the Ionic Rubber

First, bis(trifluoromethane)sulfonimide lithium salt (LiTFSI, 99%, Aladdin reagent company) was dispersed in tributyl citrate (TBC, 98%, Aladdin Reagent Company) by sonication for 0.5 h at various ratios to form organic electrolyte solutions with mass fractions of 5%−20%. PDMS (Sylgard 184, Dow Corning) was prepared by mixing the base and curing agent at a 10:1 weight ratio. The pre‐prepared organic electrolyte solution was mixed with PDMS in a certain proportion, and then a certain proportion of fumed silica (Silica, fumed, Hydrophilic, specific surface area: 400 m^2^ g^−1^, Aladdin Reagent Company) was added and stirred thoroughly with a glass rod for 10 min to obtain the ionic rubber precursor. Finally, according to the experimental requirements, the ionic rubber precursor was poured into the target mold and placed in the oven at 80 °C for 1 h to obtain the corresponding 3D shape of ionic rubber. The detailed preparation process of ionic rubber is shown in Figure S2a (Supporting Information).

### Preparation of the 3D Electrode Array

First, the structural component configured with patterned grooves and through holes was prepared by a 3D printer (Form 2, Formlabs Inc.), where the grooves and through holes were reserved on the structural component as electrode space. Second, the uncured liquid conductive silver paste was scraped onto the structural component so that the silver paste could fill the grooves and through holes of the structural component and placed in an oven at 80 °C for 2 h to make the silver paste completely cured. Finally, the structural component was ground with low and high mesh sandpaper, respectively, and the silver paste outside the structural component grooves and through holes was removed, while the silver paste inside the grooves and through holes was retained due to the protection of the structural component, resulting in a 3D electrode array.

### The Bonding and Assembly of the SES

The flexible printed circuits (FPC) with antennae‐like electrodes used to draw out the electrodes of the 3D electrode array were fabricated according to the conventional photolithography‐etching process from a third‐party organization (Shenzhen Liansheng electronic industry Co., LTD.). The 3D printed base used to connect the FPC to the 3D electrode array was prepared using a 3D printing process. The FPC was attached to a 3D printed base with a bayonet, which can be directly inserted into the 3D electrode array and fixed, and the conductive silver paste was applied to the interface between the 3D electrode array electrode and the FPC electrode and cured. Finally, the structural electronic skin was assembled by snapping the ionic rubber into the 3D electrode array.

### The Characterization of the Ionic Rubber

The morphology of the ionic rubber was characterized using SEM (Sigma 300, Zeiss). The Young's modulus of the ionic rubber was tested using a motorized force tester (ESM 303, Mark‐10) following the standard GB/T 528–2009/ISO 37:2005. The experimental setup for Young's modulus test of the ionic rubber can be found in Figure S10 (Supporting Information). The test sample was prepared into a dumbbell shape with a length of 50 mm and a thickness of 2 mm. Figure S11 (Supporting Information) shows the stress‐strain curves of ionic rubber in three Young's modulus test experiments with a weight ratio of PDMS to liquid ions of 2:1 and a silica content of 15% (compared to the weight of PDMS polymer). The UACs of the ionic rubber were measured by an Inductance‐Capacitance‐Resistance (LCR) digital bridge (TH2829C, Tonghui Inc.) at 1 kHz and a sine input with a peak voltage of 1 V (the rationale for choosing the 1 kHz test signal frequency has been included in Figure S12, Supporting Information). The sample was prepared into a film with a thickness of 1 mm, sandwiched between two electrodes with an area of 1 cm^2^. The motorized force tester compresses the sample at a rate of 0.5 mm min^−1^ and monitors the capacitance between the two electrodes until it reaches a maximum value, which was considered to be the UAC of the ionic rubber. The measurement setup of the UAC of the ionic rubber can be found in Figure S13 (Supporting Information).

### The Characterization of the SES

The capacitance‐pressure curves of the SES were characterized by measuring the capacitance of the device using an LCR digital bridge under external pressure applied by the motorized force tester, where the sensing unit area of the SES was 1 cm^2^. The scanning frequency and voltage were set to 1 kHz and 1 V, respectively. The response/reset time and the repeatability of the SES were measured by applying a periodic pressure of 1 and 10 Hz, respectively, by a piezoelectric actuator (PB4NB2S, Thorlabs Inc.). The signal generator (AFG1022, Tektronix) generates a periodic square wave with an amplitude of 1 V, which was then amplified by a power amplifier (ATA‐2022H, Xi'an Aigtek Electronic Technology Co., Ltd.) to a level of 100 V and applied to the piezoelectric actuator. The readout circuitry for single point SES is shown in Figure S14 (Supporting Information). The readout circuitry for the SES sensing array is shown in Figures S15 and S16 (Supporting Information), which was mainly composed of column selection unit (MUX), data acquisition unit (ADC), digital to analog converter (DAC), and control unit (FPGA).

## Conflict of Interest

The authors declare the following competing financial interest(s): Y.C. and T.P. are involved startup companies that are developing wearable sensing technologies. No potential conflicts of interest exist for the other authors.

## Supporting information

Supporting Information

Supplemental Video 1

## Data Availability

The data that support the findings of this study are available from the corresponding author upon reasonable request.
