# Supplementary material for: Structural Electronic Skin for Conformal Tactile Sensing
Source: Adv Sci (Weinh). 2023 Sep 22;10(33):2304106. doi: 10.1002/advs.202304106 (PMC10667827; doi:10.1002/advs.202304106)
Supplement: Supplementary file 1 — Supporting Information [file ADVS-10-2304106-s001.pdf]

ADVANCED SCIENCE
Open Access

Supporting Information

for *Adv. Sci.*, DOI 10.1002/advs.202304106

Structural Electronic Skin for Conformal Tactile Sensing

Sen Li, Jiantao Huang, Meilan Wang, Ka Deng, Chenhui Guo, Bin Li, Yu Cheng, Hongyan Sun, Hong Ye, Tingrui Pan and Yu Chang**

Supporting Information

Structural Electronic Skin for Conformal Tactile Sensing

Sen Li, Jiantao Huang, Meilan Wang, Ka Deng, Chenhui Guo, Bin Li, Yu Cheng, Hongyan Sun, Hong Ye, Tingrui Pan, and Yu Chang**

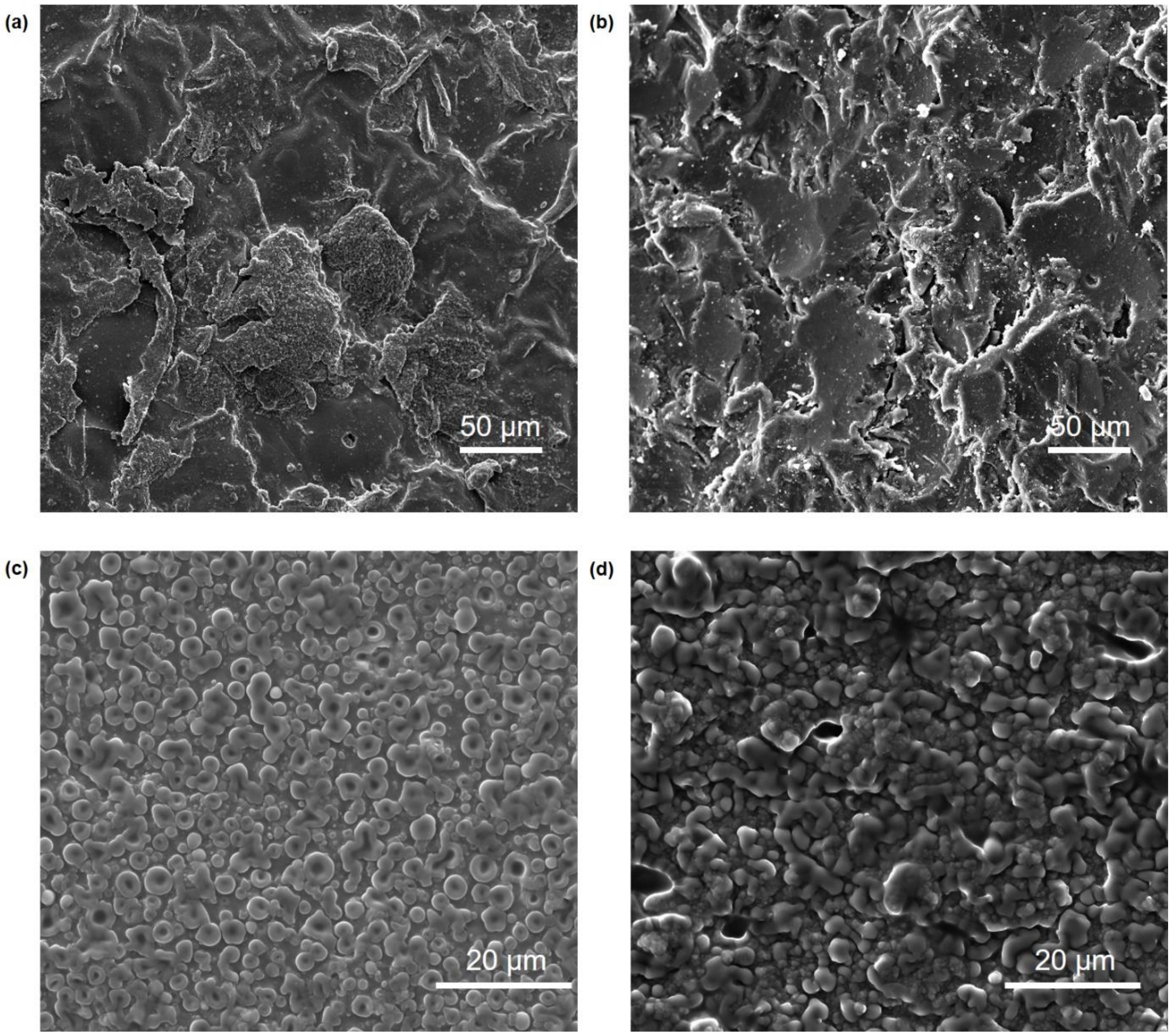

Figure S1. (a) The SEM image of ionic rubber surface; (b) the SEM image of the steel mold surface, which is processed by sand blast; the SEM images of the cross section of the ionic rubber (c) without and (d) with fumed silica added;

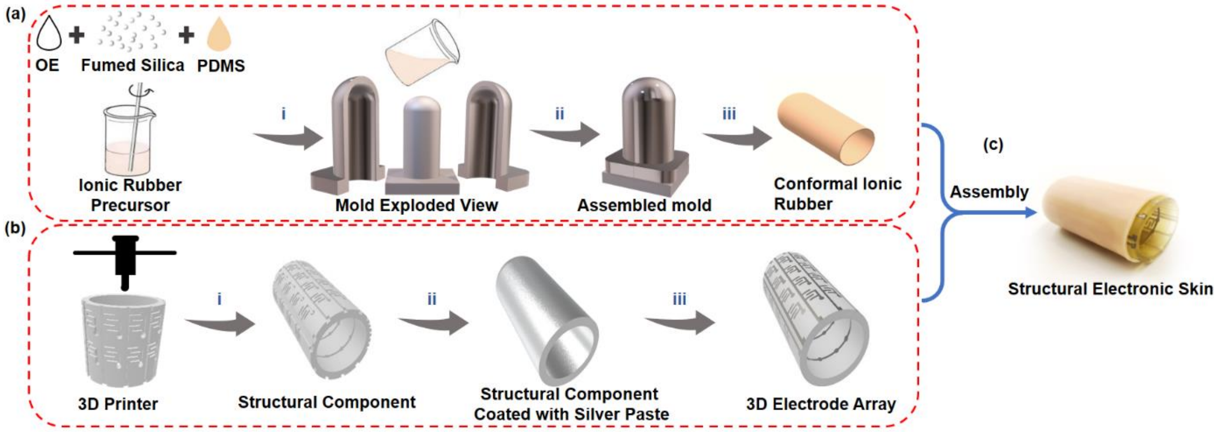

Figure S2. (a) Conformal ionic rubber preparation process flow chart (i: Preparing Ionic Rubber Precursor, ii: Fill the Mold with Ionic Rubber Precursor and Cure, iii: Demolding); (b) 3D electrode array preparation process flow chart (i: Preparing structural component by 3D printing, ii: Scrape and cure silver paste on structural component, iii: remove of redundant silver paste); (c) photo of assembled structural electronic skin.

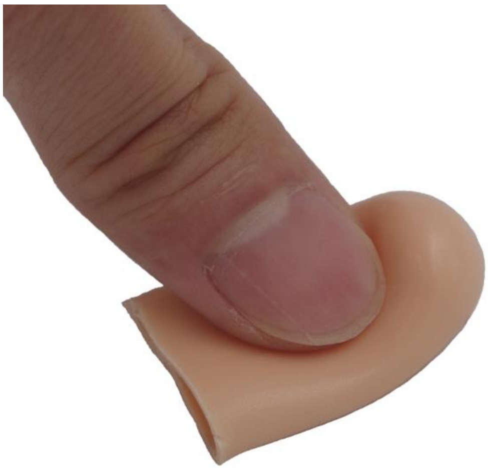

Figure S3. The elasticity of the ionic rubber.

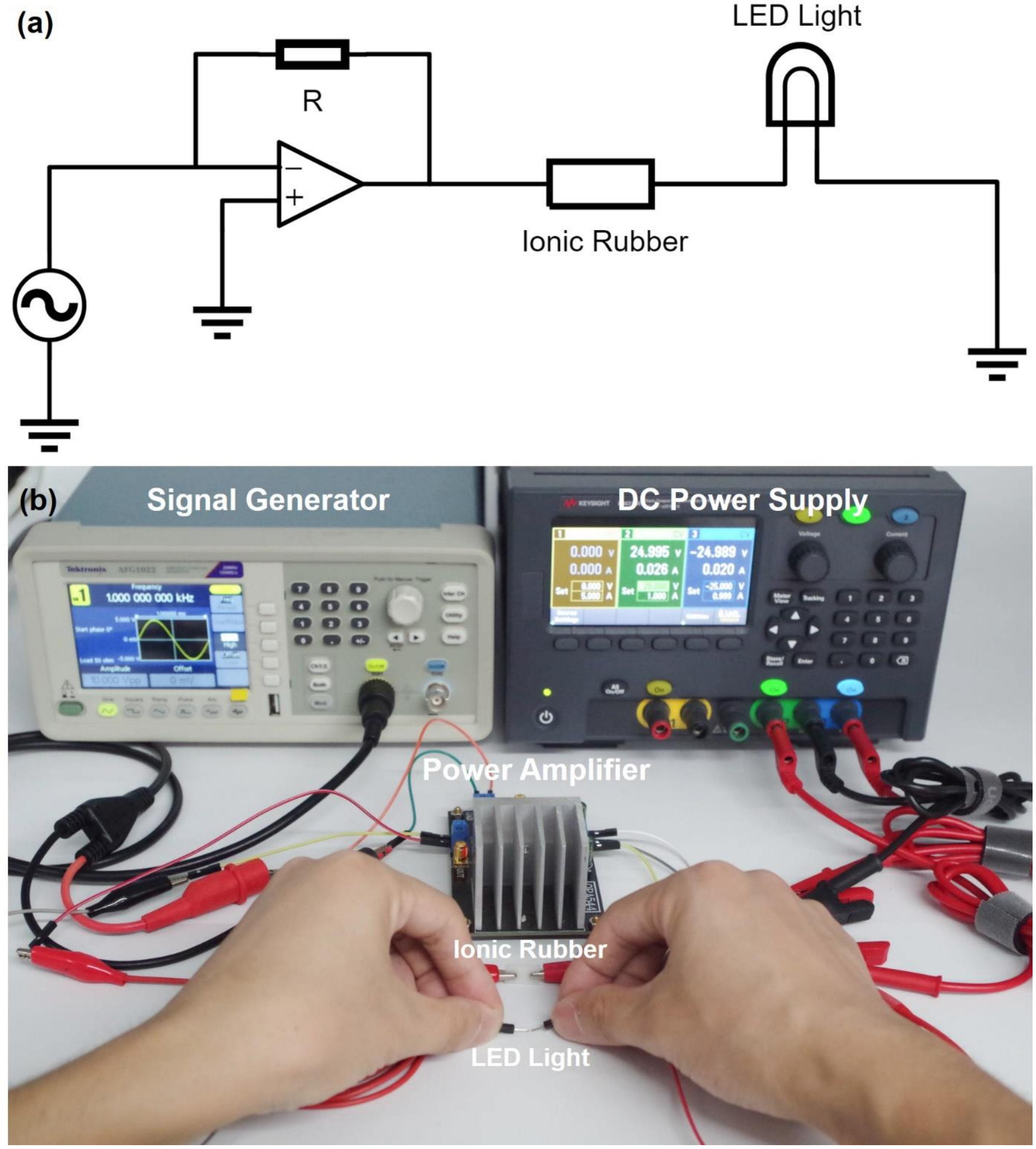

Figure S4. (a) Ionic rubber conductivity test circuit diagram; (b)Experimental setup for conductivity test of the ionic rubber.

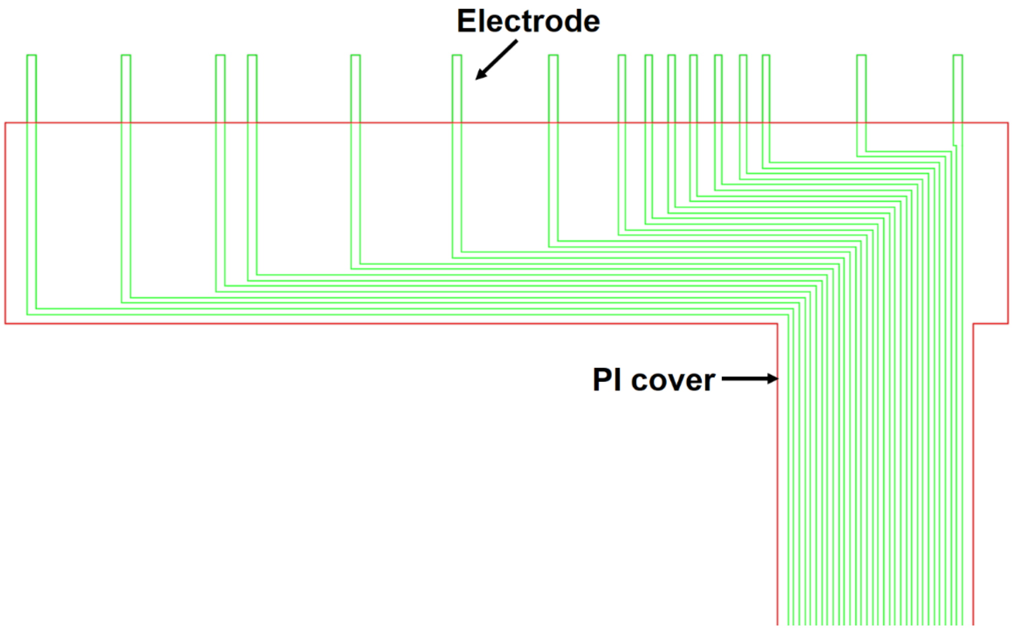

Figure S5. Flexible printed circuit (FPC) design schematic for bonding the 3D electrode array and the signal readout circuit.

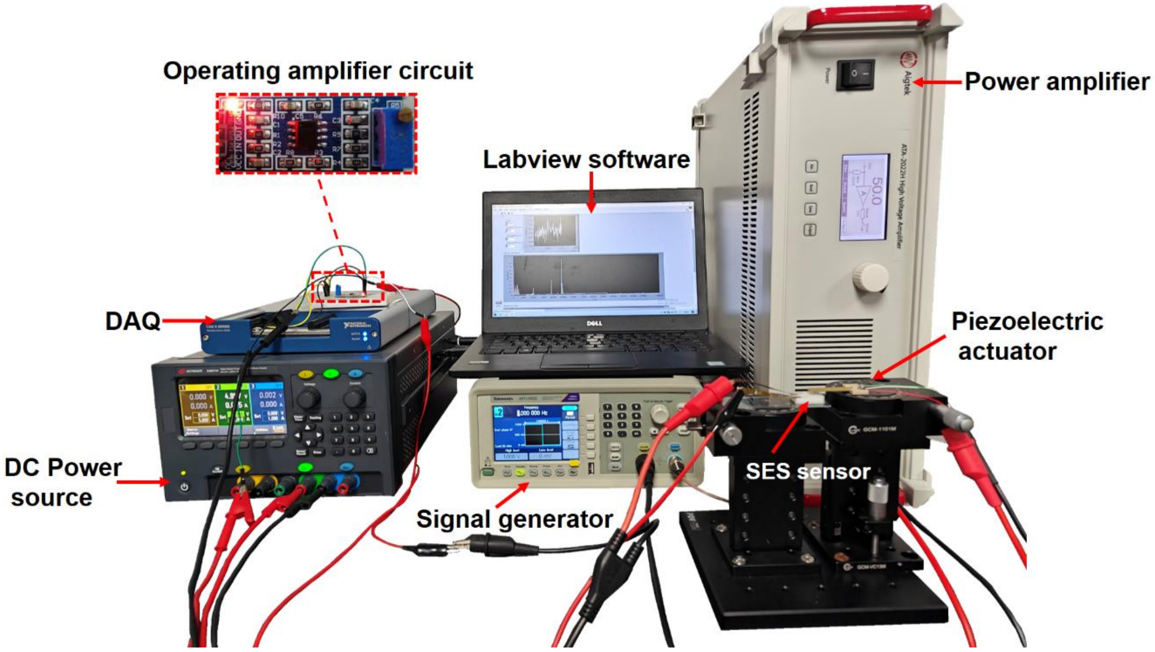

Figure S6. Measurement setup of the response time and repeatability of the SES.

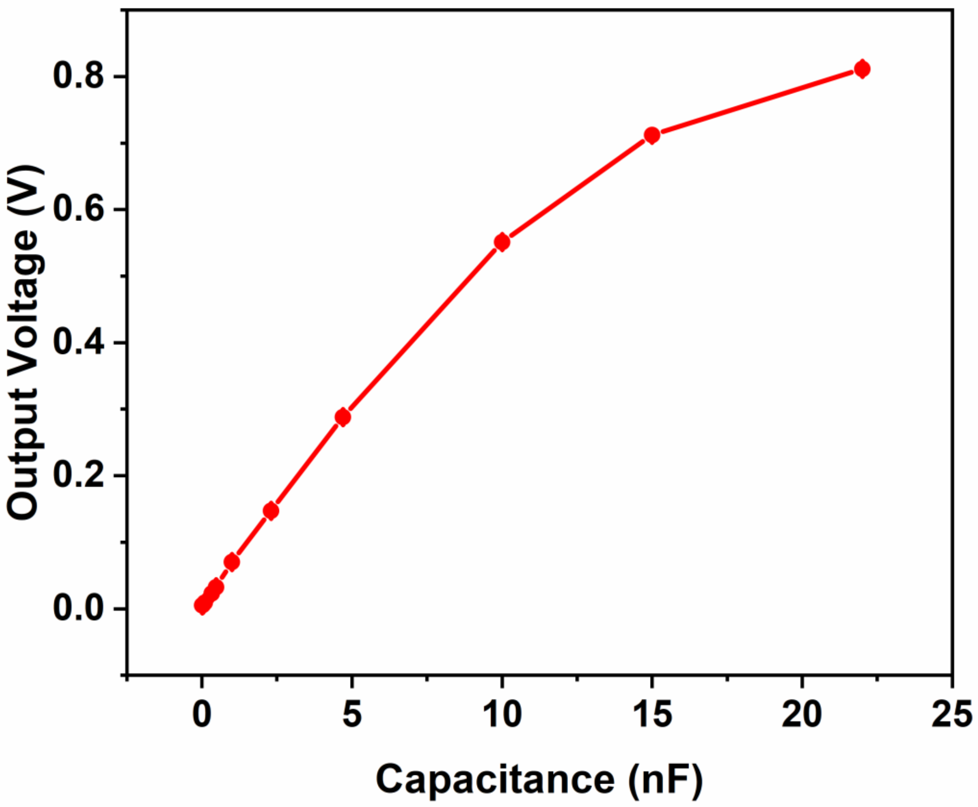

Figure S7. The correlation between the output voltage signal and the capacitance signal.

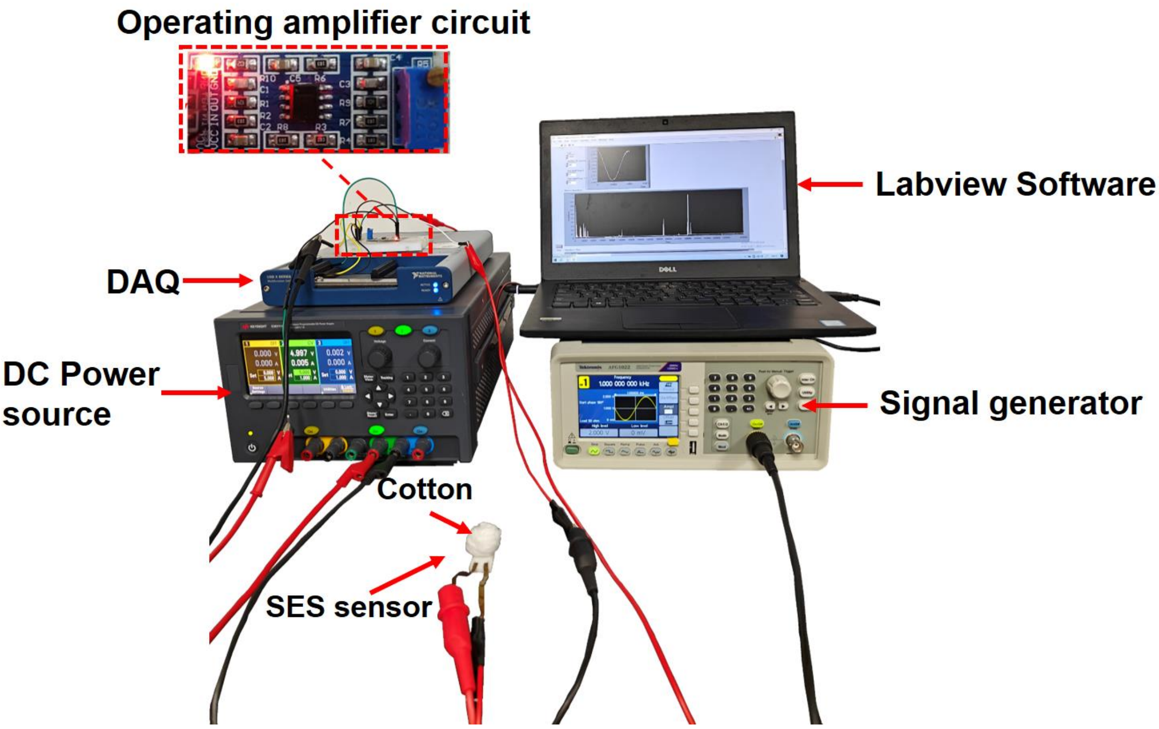

Figure S8. Measurement setup of the minimal pressure resolution of the SES.

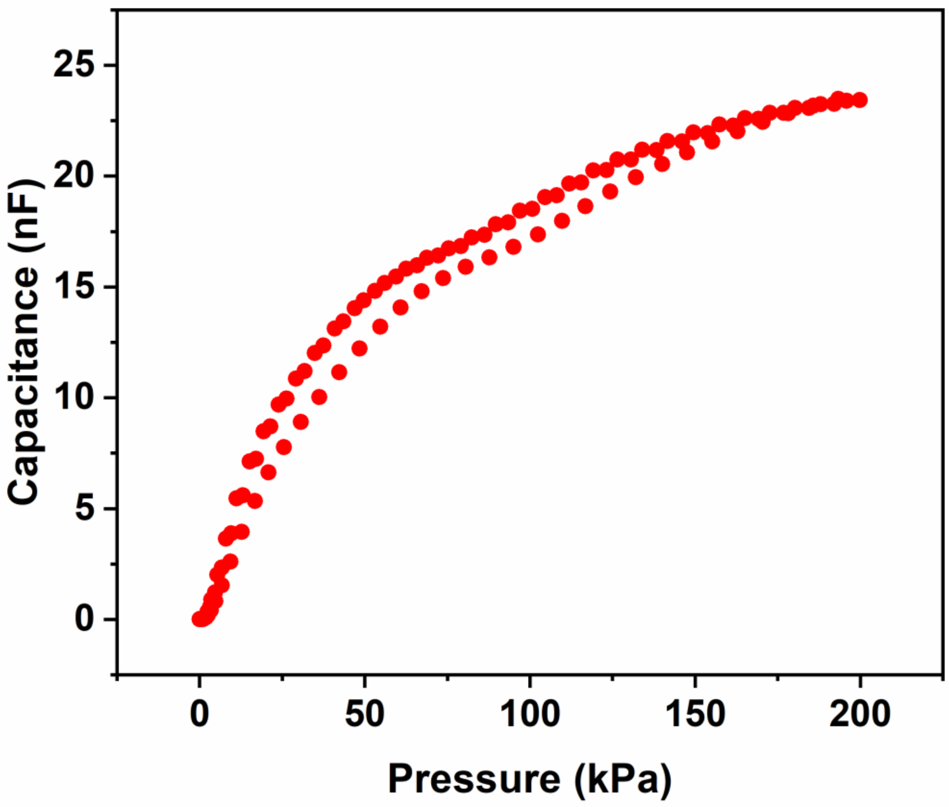

Figure S9. The capacitance-to-pressure response of the sensor based on the designed electrode size(4×4mm) in the 3D printed fingertip.

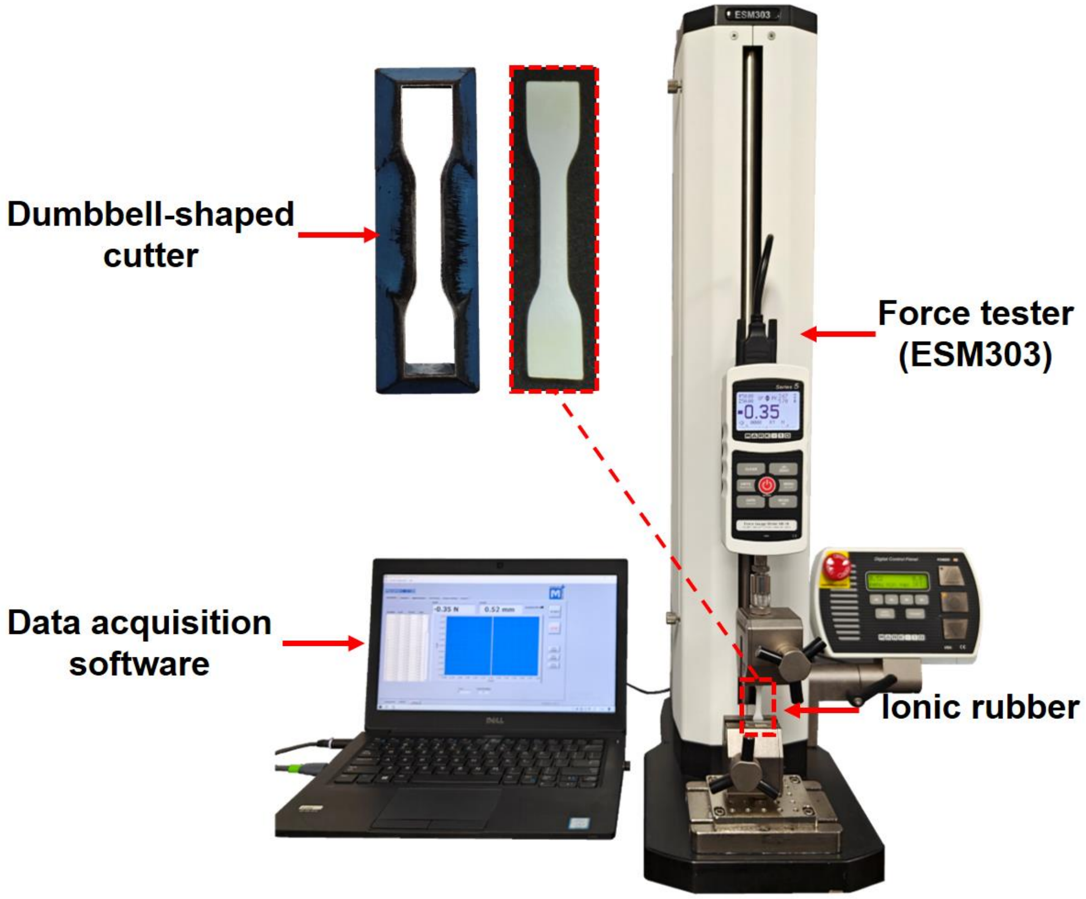

Figure S10. Experimental setup for Young's modulus test of the ionic rubber.

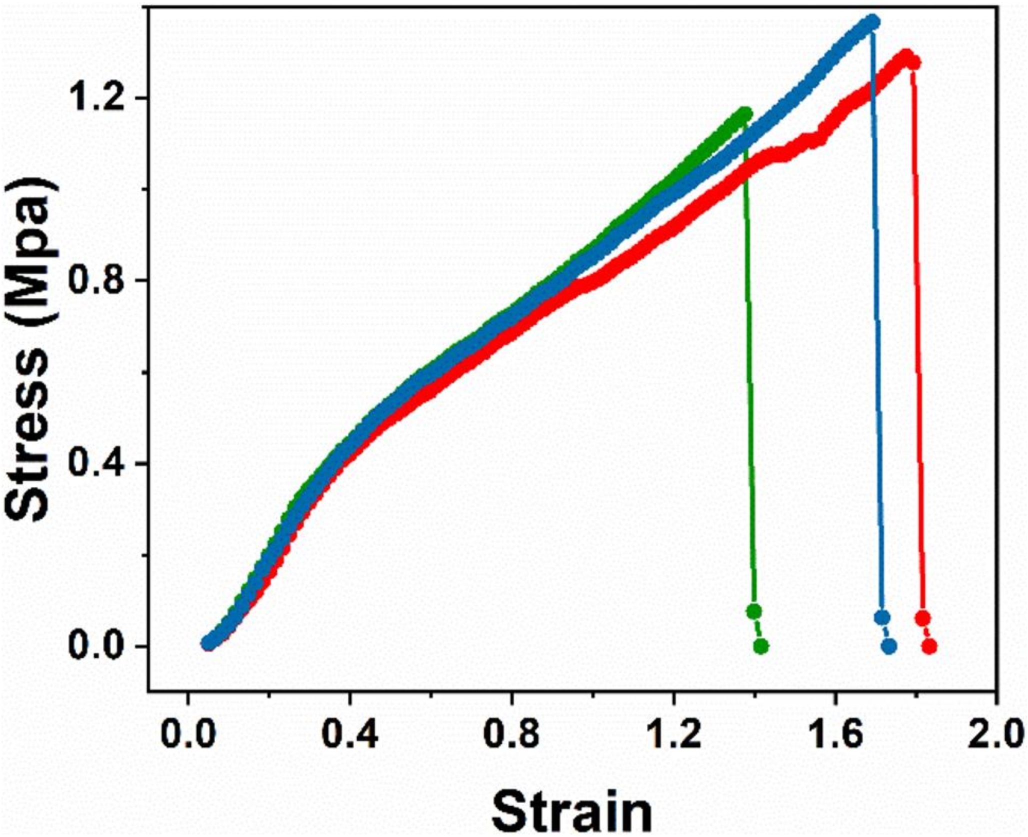

Figure S11. Stress-strain curves of ionic rubber in which the weight ratio of PDMS to liquid ionics is 2:1 and the silica content is 15% (compared to the weight of PDMS polymer).

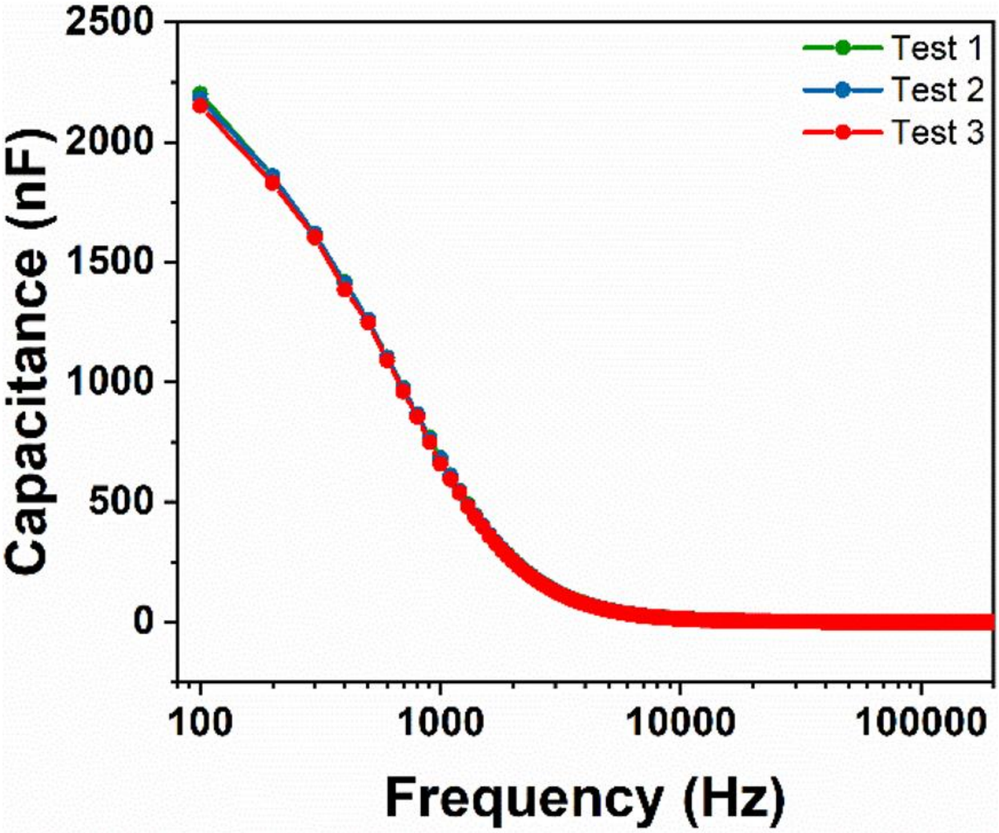

Figure S12 Three measurements of the UAC of ionic rubber at different driving frequencies.

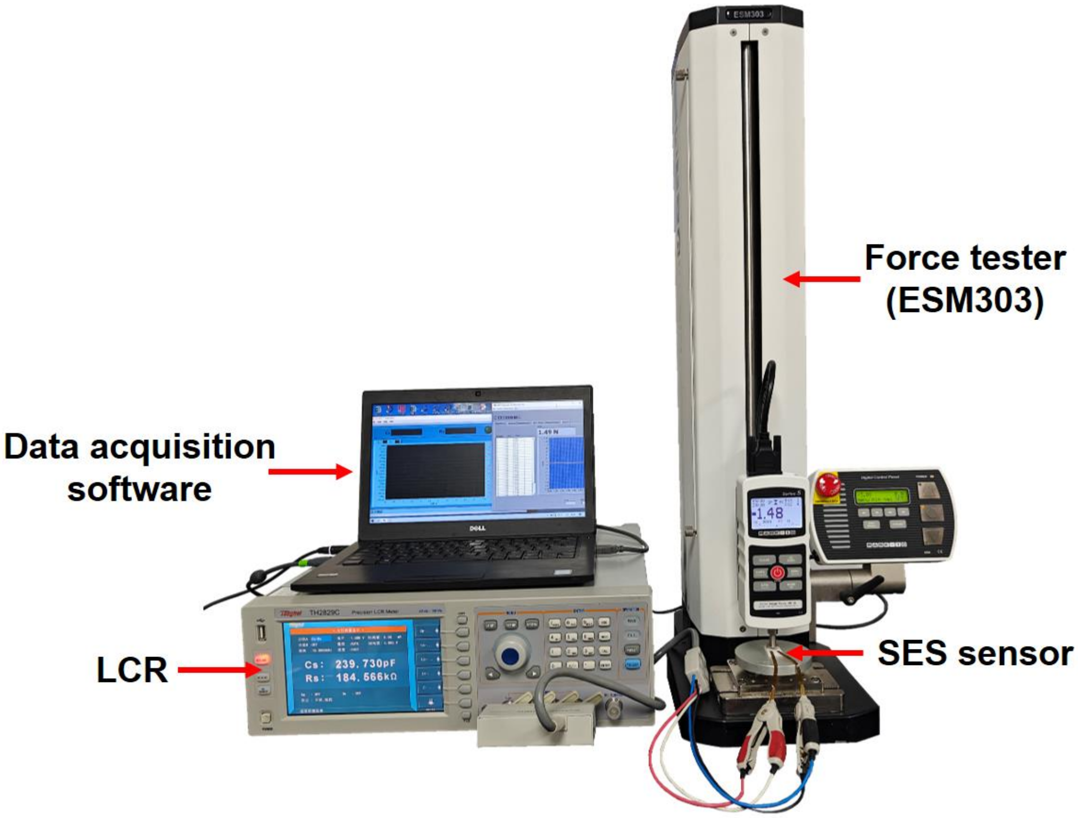

Figure S13. Measurement setup of the capacitance pressure response and UAC of the SES.

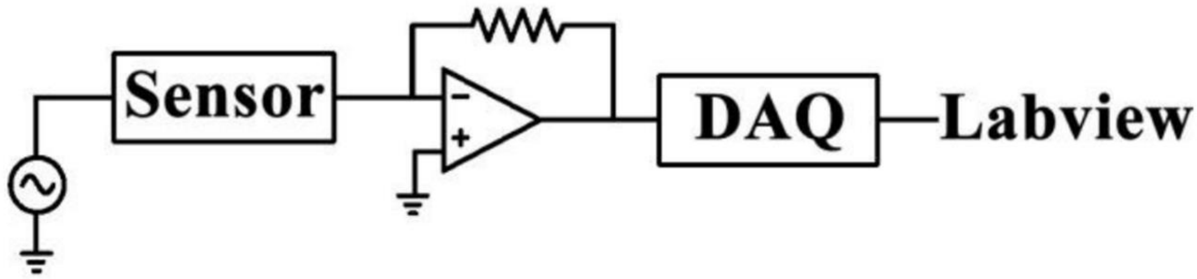

Figure S14. The readout circuitry for single point SES.

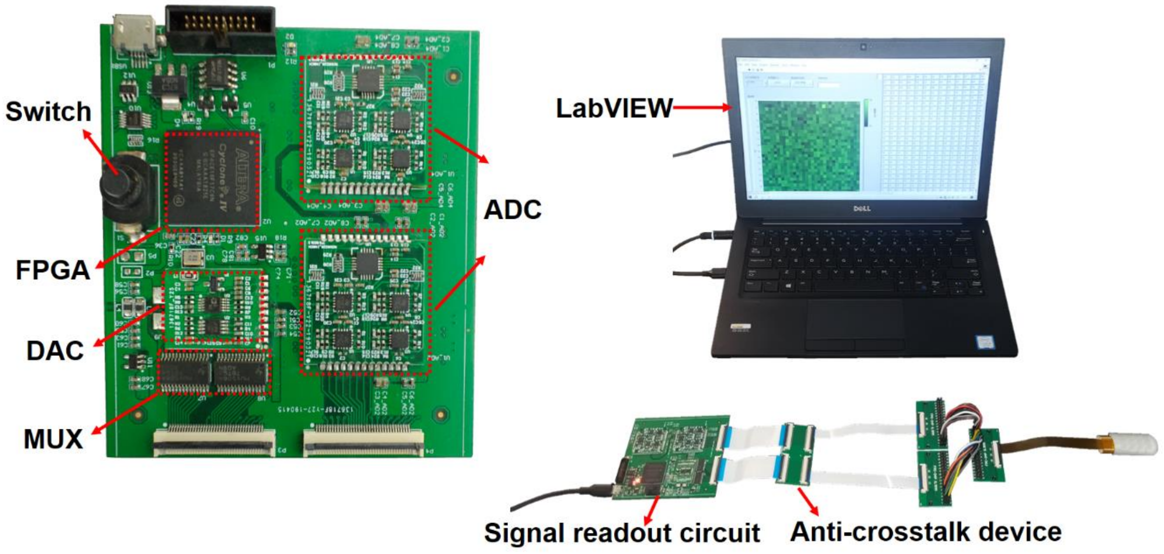

Figure S15. Readout circuitry of the sensing array and the Labview software for recording and displaying the tactile images.

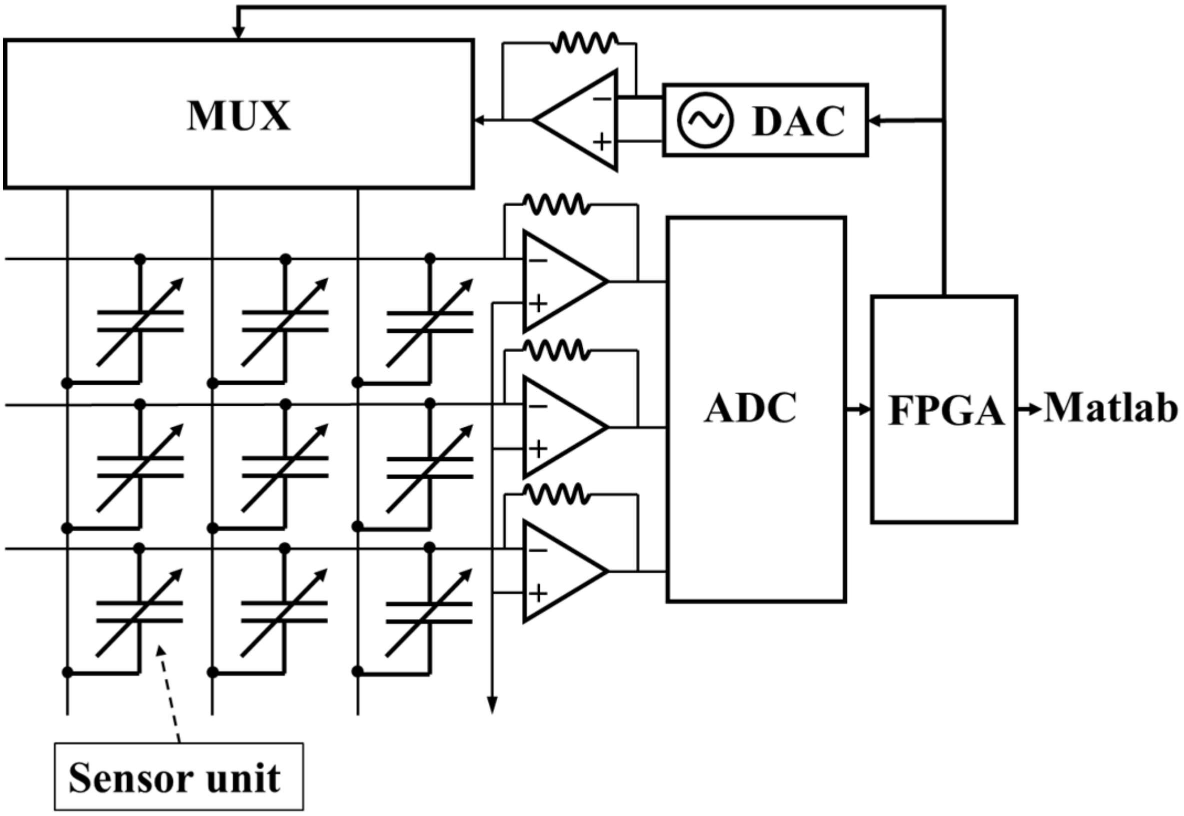

Figure S16. The principal of the readout circuitry for matrix sensing of the SES array.
